# Application of natural products combined with plant-derived extracellular vesicles in cancer therapy

**DOI:** 10.3389/ebm.2026.11329

**Published:** 2026-10-09

**Authors:** Ruyue Wang, Menglin Ren, Yuanjian Xu, Yan Sun, Zenghui Xu

**Affiliations:** 1 Shanghai Mengchao Cancer Hospital & School of Medicine, Shanghai University, Shanghai, China; 2 Shanghai Cell Therapy Group Company Limited, Shanghai, China; 3 Shanghai Cell Therapy Research Institute, Shanghai, China; 4 Medical College of Shanghai University, Shanghai, China

**Keywords:** antitumor activity, drug delivery, immunology, phytochemicals, plant-derived nanovesicles

## Abstract

Cancer remains a leading cause of mortality worldwide. The success of plant-derived chemotherapeutic agents, such as paclitaxel and vinblastine, underscores the significant potential of plant-derived natural products (PDNPs) in oncology. Despite this success, the poor solubility, metabolic instability, and nonspecific distribution of many PDNPs pose a fundamental barrier to their full clinical potential. Plant-derived extracellular vesicles (PDEVs) have garnered considerable attention as biocompatible nanocarriers, and numerous reviews have examined their biological activities and delivery applications. However, few have systematically addressed their synergistic combination with PDNPs in cancer therapy. This review distinguishes itself by centering on the synergistic interplay between PDNPs and PDEVs as an integrated therapeutic strategy. Specifically, we examine: (1) how PDEVs enhance the bioavailability and anticancer efficacy of PDNPs by improving their solubility, stability, and targeted delivery, and (2) how the intrinsic antitumor activity of PDEVs complements that of PDNPs to achieve enhanced therapeutic efficacy and reduced systemic toxicity compared with either agent alone. By integrating current evidence on the combined use of PDNPs and PDEVs, this review aims to enhance the field’s understanding of the progress made by PDNPs and PDEVs in cancer therapy, clarify their mechanisms of action, and identify the critical challenges facing the current research stage.

## Impact statement

This review is organized around the synergistic PDNPs-PDEVs axis. Plant-derived extracellular vesicles (PDEVs) are shown to act simultaneously as intrinsic antitumor agents and as delivery nanocarriers that overcome the bioavailability and targeting barriers of plantderived natural products (PDNPs). This work fosters interdisciplinary integration across plant biology, nanomedicine, and oncology, accelerating the clinical adoption of PDNP-PDEV-based anticancer strategies.

## Introduction

Cancer remains a leading cause of death worldwide, with over 10 million deaths each year [1]. Despite advances in therapy, tumor heterogeneity and drug resistance limit long-term success, driving continued interest in PDNPs as a source of novel anticancer agents.

Natural products have been pivotal to anticancer-drug discovery: approximately 55% of approved agents have been natural or derived from them since 1983, with plants contributing 60% of these natural-product-derived agents [2, 3]. Most PDNPs exhibit poor water solubility, which directly results in reduced bioavailability and an erratic absorption profile[4]. Chemical instability under physiological conditions and susceptibility to enzymatic degradation often prevent therapeutic concentrations from reaching tumor sites [5]. Additionally, their non-specific tissue distribution often causes severe adverse reactions, which further compounds the challenges faced by PDNPs in oncology [6].

PDEVs are nanoscale particles encapsulated within lipid bilayers. These vesicles contain a complex cargo of bioactive molecules, including proteins, lipids, nucleic acids, and secondary metabolites, all of which reflect the plant of origin [7]. A growing body of evidence underscores the therapeutic potential of PDEVs in diverse diseases, including cancer, inflammatory disorders, and intestinal diseases, while consistently demonstrating a favorable safety profile [8–10]. The anticancer efficacy of PDEVs operates through a dual mechanism. First, as intrinsic therapeutics, they are enriched with plant-specific bioactive molecules (such as phenolic compounds, flavonoids, and miRNAs) that can modulate key oncogenic pathways such as PI3K/AKT and MAPK [11, 12], thereby inhibiting proliferation, inducing apoptosis, and suppressing metastasis; second, their innate nanostructure and biocompatibility render them ideal candidates for engineered drug delivery systems [13].

While an increasing number of reviews have covered various facets of PDEV biology, most are organized around PDEVs themselves as the central subject, rather than systematically examining the interplay between PDEVs and PDNPs in the context of cancer therapy. Specifically, the synergistic relationship between PDEVs and PDNPs—which encompasses both PDEVs-mediated delivery of PDNPs and the complementary therapeutic effects of the two—has not yet been synthesized as a coherent theme in the current review landscape. Building on this gap, this review provides a systematic analysis centered on the synergistic application of PDNPs and PDEVs in cancer therapy and is organized as follows. First, it summarizes the antitumor properties and molecular mechanisms of PDEVs, which lay the biological foundation for understanding their complementary and synergistic effects with PDNPs. Second, it examines how PDEVs serve as delivery vectors to improve the bioavailability and anticancer efficacy of PDNPs, representing a core dimension of their synergy. Finally, it discusses the translational prospects and clinical barriers of PDNPs–PDEVs combination systems. The overall objective is to provide a focused reference for the development of PDNPs–PDEVs-based combinatorial cancer therapeutics.

## Biogenesis and isolation of PDEVs

### Classification and biogenesis of PDEVs

According to biogenesis, PDEVs can be classified into different subtypes: exosomes (50–150 nm), microvesicles (100–1000 nm), migrasomes (up to 3000 nm) and apoptotic bodies (diameter >1,000 nm). There are three main pathways for the biogenesis and release of PDEVs: the endosomal pathway, vacuole pathway, and exocyst-positive organelle (EXPO) pathway (Figure 1) [14, 15]. The endosomal pathway is the main pathway and is highly similar to the biogenesis of ADEVs [16, 17]. Cytoplasmic membrane invagination by endocytosis-associated proteins forms early endosomes, and endosomal sorting complex required for transport causes the deformation of the membrane, allowing the sorting of cargo molecules within endosome [18, 19]. The early endosome matures and interacts with the trans-Golgi network to form the MVB (multivesicular body). A small fraction of MVB is degraded in lysosomes, whereas the majority of MVB can release intraluminal vesicles outside the plasma membrane to form PDEVs [20, 21]. Such EVs, originating from internal cellular compartments and released via MVBs, are termed exosomes. PDEVs can also be generated through the vacuole pathway. In this process, vacuoles merge with MVB, encapsulate intraluminal vesicles, and are subsequently released upon fusing with the plasma membrane. These vesicles, released directly from the plasma membrane, are referred to as ectosomes [22]. PDEVs can be produced by the plant-specific EXPO, which possesses a double-membrane structure akin to autophagosomes. EXPO fuses with the plasma membrane to release single-membrane vesicles into the cell wall, termed as EXPO microvesicles [21, 23]. Migrasomes, which originate from the cytoplasmic membrane surface [24]. Additionally, when plant cells undergo programmed cell death, the cell membrane buds outward to enclose the cytoplasm and degraded organelles. This process forms apoptotic bodies through constriction. These structures can then be released into the extracellular space, serving as an important source of PDEVs.

**FIGURE 1:**
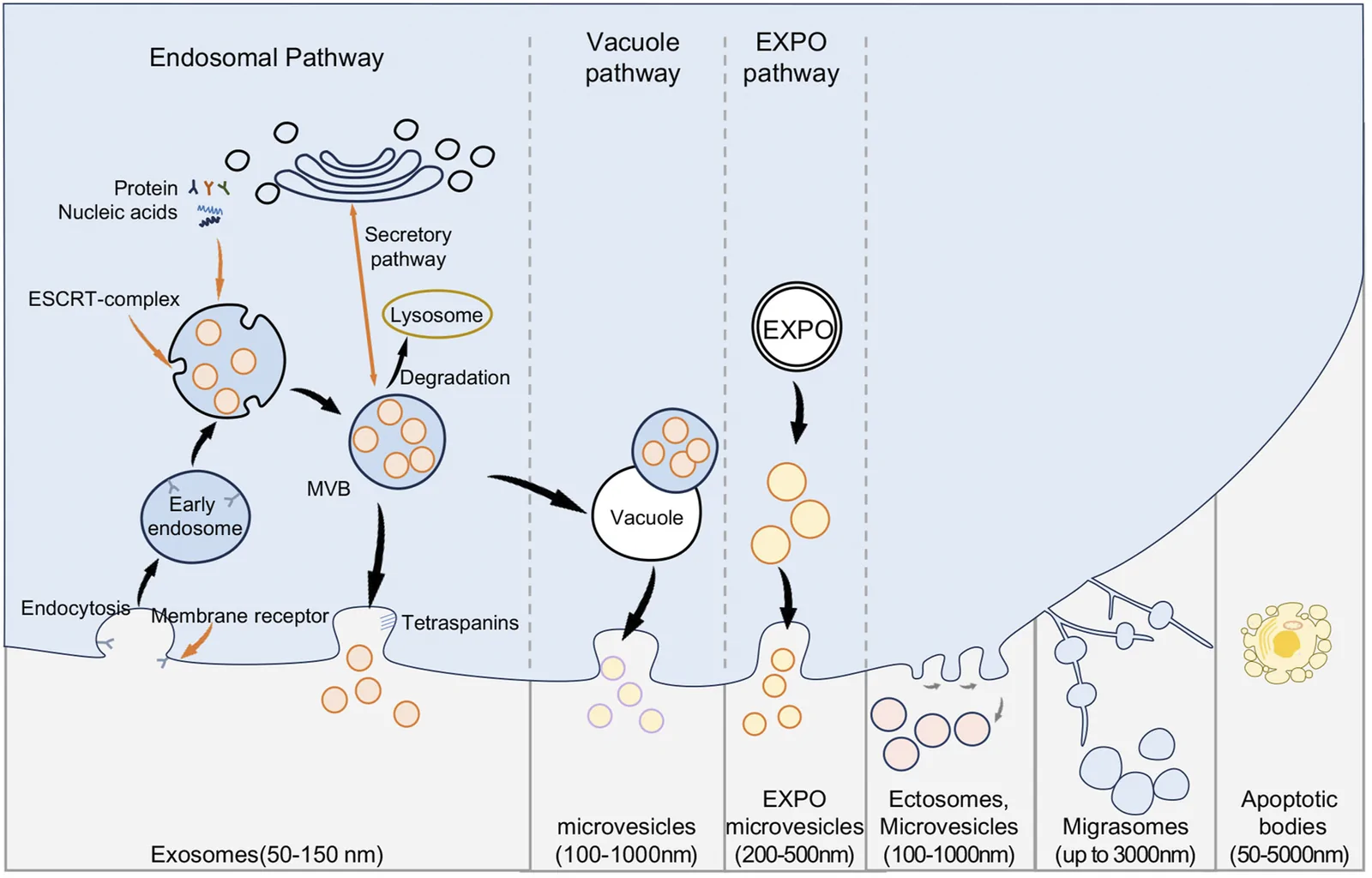
Biogenesis and secretion of PDEVs. PDEVs can be secreted from plant cells through multiple path-ways: the Endosomal Pathway, the vacuolar pathway, and the EXPO pathway. Plant microvesicles originate from the plasma membrane and are subsequently released extracellularly. Migrasomes, the vesicles formed at the leading edge and junctions of contractile fibers during cell migration. Apoptotic bodies, during programmed cell death, the cell membrane undergoes inward invagination and segmentation, encapsulating cytoplasmic materials, including DNA and organelles, to form vesicle-like structures. ESCRT: endosomal sorting complex required for transport.

Among the various types of vesicles, exosomes have attracted significant attention because they are believed to possess unique biological functions. However, due to the current lack of uniform standards for their isolation and identification, the “exosomes” reported in many studies are actually mixed populations of various vesicle subtypes.

### Isolation and identification of PDEVs

The isolation of PDEVs presents a unique challenge not encountered with ADEVs: the plant cell wall. Consequently, the PDEVs isolation process must address two consecutive challenges: first extracting apoplastic fluid with minimal contamination, and then purifying vesicles from this complex mixture [25, 26]. Milling and juicing [27], vacuum infiltration and enzymatic cell wall digestion [26] represent the three main pretreatment strategies to extracting apoplastic fluid, each with distinct advantages and drawbacks (Table 1). No single approach can simultaneously maximize yield, purity, and functional integrity. The choice of pretreatment directly affects the properties and bioactivity of PDEVs, making the selection of an appropriate pretreatment method the first critical challenge in PDEVs applications.

**TABLE 1:** Pretreatment methods for isolating PDEVs from plant tissues.

Technology	Working principle	Advantages	Limitations	References
Milling and juicing	Physical shearing and crushing of tissues to release content	Rapid; simple; high yield	Low purity due to massive cellular debris	[26, 131, 132]
Vacuum infiltration	Vacuum-assisted buffer infiltration into apoplast, followed by pressure-gradient extraction of apoplastic fluids	Preserves vesicle integrity; avoids intracellular contaminants	Low yield due to the cell wall barrier	[133]
Enzymatic digestion	Degraded cell walls with enzymes	High purity; high yields	Introduces enzyme impurities; requires parameter optimization; higher cost	[26]
Root exudate	Purification from the root exudates of hydroponic plants	High purity	Applicable to specific plants only; low yields; limited subtype of PDEVs	[134]

Once the pretreated crude extract is obtained, PDEVs can be isolated. Ultracentrifugation (UC), which employs high centrifugal forces (such as 100,000 × g), is a widely used method that balances efficiency and purity [28]. UC is suitable for basic research and small- to medium-volume samples, but its scalability for large volumes is limited. Size exclusion chromatography (SEC) utilizes differences in the hydrodynamic radius of PDEVs relative to other biological contaminants to achieve high-purity isolation. A key advantage of SEC is its ability to preserve the structural and functional characteristics of PDEVs while minimizing the co-isolation of non-vesicular components. However, larger sample volumes may require pre-filtration to avoid column clogging. Ultrafiltration (UF) separates particles based on size exclusion through semipermeable membranes, permitting the passage of small molecules and solvents while retaining larger particles such as EVs. Tangential flow filtration (TFF) has gained prominence in EVs isolation due to its continuous flow design, which reduces pore clogging and filter cake formation common in conventional dead-end filtration methods [29, 30]. Although TFF has demonstrated superior yield (five-fold over UC) for EVs from liposuction fluid [29], comparable data for PDEVs are currently lacking, highlighting the need for plant-specific validation. Immunoaffinity Capture captures EVs by recognizing highly specific antigens on their surface. The extravesicular loop domain of Tetraspanin 8 (TET8) is extensively exposed on the surface of *Arabidopsis thaliana* L. EVs. Based on this characteristic, researchers have successfully isolated TET8-positive EVs using a TET8 antibody [31]. The limitations of this method include the high cost of antibodies and the potential impact of antibody elution on the structural integrity of EVs [32]. Most of the above-mentioned isolation methods, originally developed for ADEVs, have not yet been established as a standardized protocol for PDEVs. Single methods often struggle to combine high recovery with high purity. Therefore, selecting a multi-method strategy based on application objectives may become the optimal solution. A study separately compared UC, UC-SEC, and UC-dgUC separated EVs from pleural effusions, identified that the UC-SEC yielded the purest EVs with the highest protein content [33]. Chen et al. proposed combining TFF with SEC to enhance purity: TFF performs initial concentration, followed by SEC for size-based refinement [180]. In addition, microfluidics enables efficient and gentle purification of EVs by precisely controlling fluid flow and pressure parameters, minimizing damage to vesicle structure [30], and shows great potential for high separation purity and good reproducibility, which may become an important technology for the validation and standardization of PDEVs.

Beyond conventional approaches, innovative techniques based on surface charge differences have also emerged for PDEVs isolation (Table 2). Yang et al. used an electrophoresis-dialysis method to isolate EVs from lemon juice, where an electric field, biological contaminants (proteins and nucleic acids) migrated out of the dialysis bag [34]. Khan et al. introduced a reciprocating free-flow isoelectric focusing method, which was validated on ADEVs and could potentially be adapted for PDEVs [35]. Giancaterino et al. proposed that integrating electrophoretic and ionic membrane processes with modular design enables continuous, high-throughput EVs separation [30]. However, these approaches remain exploratory, facing challenges such as voltage-induced structural damage, low yield, and limited scalability to GMP standards.

**TABLE 2:** The isolation techniques of PDEVs.

Technology	Working principle	Advantages	Disadvantages	References
Density ultracentrifugation	Stepwise centrifugation to separate particles	Gold standard method Moderate purity Low cost Suitable for small and medium sample sizes	Risk of damage to PDEVs activity Requires specialised equipment Time-consuming Difficulty in scaling up production	[25, 135]
Density Gradient UC	Sucrose/iodide gradient separation	Higher purity than UC	Risk of damage to PDEVs activity Low yield Requires specialised equipment Complex operation Difficulty in scaling up production	[32]
Size exclusion chromatography (SEC)	Separates particles by hydrodynamic size as they pass through a porous resin	Preserves vesicle structure High purity	Requires specialised equipment Time-consuming Low throughput	[136]
Ultrafiltration Tangential flow filtration	Membrane filtration separation, TFF uses tangential flow to minimize membrane clogging	Preserves vesicle structure High purity Simple operation (UF) Scalable and suitable for industrial scale (TFF)	Membrane fouling leads to low yields A large sample size can be time-consuming Requires optimization of membrane pore size (TFF)	[30, 137]
Polymer precipitation	Uses polymers (like PEG) to dehydrate and precipitate PDEVs	Simple operation Low cost No special equipment Suitable for large volumes	Low purity due to non-specific co-precipitation	[25]
Immunoaffinity capture	Antibody-specific binding	High specificity and purity for subpopulations	High cost, limited to known markers	[32]
Electric charge-based Isoelectric focusing Electrophoresis coupled dialysis (ELD) Anion-exchange chromatography (AEC)	Separates particles based on surface charge differences	High resolution High sensitivity Does not require specialized equipment (ELD) Fast method (ELD) Enable a large quantity (AEC)	A high electric field may damage PDEVs Exploration phase Open environment	[34, 35, 138, 139]

Characterization techniques after isolation are key to PDEVs applications. Based on MISEV2023 released by the International Society for Extracellular Vesicles, it is recommended to employ a five-component orthogonal technology combination for PDEVs identification. Regarding protein marker assessment, detection of at least one protein from each of the following categories is mandatory: Category 1 (transmembrane proteins), Category 2 (cytoplasmic proteins), and Category 3 (associated with the stability and function of PDEVs). Additionally, the topology of PDEVs-associated components should be assessed, that is, whether a component is luminal or on/at the surface of PDEVs.

Accordingly, we outline the major biomarkers currently under consideration (Table 3). Most work has been done in Arabidopsis and other Brassicaceae species, where the tetraspanins TET8 and TET9, the syntaxin PEN1/SYP121, and EXO70E2 (for EXPO-derived vesicles) are widely used as classical protein markers [36, 37]. Patellins (PATL1/PATL2) have been detected in sorghum, tomato roots, citrus and many other plants, and are recognized as candidate PDEVs biomarkers. Yet their co-existence within apoplastic soluble proteins reduces marker specificity. For practical use, they must be combined with other specific markers for joint validation to eliminate false positives arising from apoplastic soluble protein contamination[38, 39]. Recently, plant-specific fasciclin-like arabinogalactan proteins (FLAs) and germin-like proteins have emerged as novel PDEVs candidate biomarkers [37, 40]. However, no current candidate markers have reached the rigorous validation level of mammalian EV biomarkers. The absence of unified biomarker consensus causes prominent limitations. It hinders the cross-study comparability of experimental results. Meanwhile, overexpression dependence and the scarcity of specific antibodies further weaken experimental repeatability. Recent large-scale proteomics and phylogenetic conservation analyses based on dual reference species have established an important foundation for biomarker standardization. Nevertheless, extensive cross-species and cross-method validations are still required to form field-wide consensus. In this context, systematically identifying vesicle-conserved proteins and accurately distinguishing non-vesicle contaminant proteins can provide critical theoretical evidence for clarifying the biological functions of PDEVs.

**TABLE 3:** Potential markers of PDEVs.

Protein family	Protein subtype	Sources	Advantages and limitation	References
Transmembrane proteins				
Aquaporins	PIP12, PIP21, PIP27	Sorghum, citrus, broccoli, lemon	More conserved than TET8 proteins; their specificity for PDEVs remains unconfirmed, as these proteins also exist in other membrane structures	[40, 140, 141]
Tetraspanin	TET8, TET9	*Arabidopsis thaliana*, *Nicotiana benthamiana*, sorghum, Sunflower, lemon, kiwi pollen	Recognized as a specific marker of PDEVs; distant homologous proteins also exist in animals	[38, 40]
Type-II integral membrane protein	PEN1, PEN3	*N*. *benthamiana*	No commercial antibodies are available for native protein detection; its broad conservation across various crops requires further experimental validation	[38]
V-type proton ATPases	VATB1, VATA, VATE1	Sorghum, Sunflower, kiwi pollen, lemon	Evolutionarily conserved among plant and not plant-specific	[40]
Cytosolic proteins				
Heat shock proteins	HSP70, HSP90	Sorghum, Sunflower, lemon, kiwi pollen, sweet orange, grapefruit, bitter orange, mulberry bark	Show poor specificity for PDEVs and can only be used as auxiliary references, rather than an independent criterion for PDEVs identification	[40, 141, 142]
GAPDH	N/A	*A*. *thaliana*, *N. benthamiana*	Highly abundant in the cytosol; cannot serve as a specific PDEVs marker	[133]
Exocyst complex component	EXO70E2	*A*. *thaliana*, *N*. *benthamiana*	Specific to EXPO-derived vesicles; not representative of total PDEVs	[23, 36, 143]
Pattelins	PATL-1, PATL-2	*A*. *thaliana*, *N*. *benthamiana*, sorghum, tomato root, *Camellia sinensis*, citrus	Conserved across species; not PDEVs-specific	[38, 39]
Luminal proteins				
Calreticulin	N/A	*A*. *thaliana*, *Brassica oleracea*	Conserved across species; ER-resident protein, prone to false-positive signals	[40]
Glutathione S-transferase	GSTF, GSTU, GSTP	Lemon, citrus, grape	Commonly found in PDEVs; not PDEVs-specific and susceptible to cytosolic contamination	[65, 133, 141]
Surface-associated proteins				
Fasciclin-like arabinogalactan proteins	FLA10, FLA13	Pine callus tissue, sorghum, Sunflower, lemon	Plant-specific; potential PDEVs-specific marker; limited cross-species conservation	[40]
Germin-like proteins	GL31, GL33	Sunflower, citrus	Plant-specific; limited cross-species conservation	[40]

GAPDH: Glyceraldehyde-3-phosphate dehydrogenase; N/A: not available; ER: endoplasmic reticulum.

## Synergistic mechanisms of PDNPs–PDEVs in cancer therapy

### PDEVs improve PDNPs bioavailability and targeting

PDNPs play a crucial role in cancer therapy, acting through multiple mechanisms including antiproliferative, proapoptotic, antiangiogenic, and antimetastatic effects [41, 42]. However, their clinical translation is hampered by poor aqueous solubility, limited biodistribution, and rapid metabolic clearance, leading to low bioavailability [43]. The synergy between PDNPs and PDEVs operates at two levels: pharmacokinetic enhancement via improved delivery, and pharmacodynamic complementation via combined bioactivity. This section focuses on the first, examining how PDEVs address core pharmacokinetic limitations of PDNPs.

#### Improved aqueous solubility of hydrophobic PDNPs

Hydrophobic natural products such as curcumin, resveratrol, and quercetin typically exhibit aqueous solubilities in the microgram-per-milliliter range [44], making it difficult to achieve therapeutically relevant systemic concentrations. As natural nanoscale delivery systems, PDEVs possess an amphipathic lipid bilayer that enables efficient encapsulation of hydrophobic PDNPs through two distinct mechanisms. Highly lipophilic phytochemicals, including curcumin and resveratrol, can be incorporated into the lipid bilayer via hydrophobic interactions, while water-soluble compounds can be loaded into the aqueous inner lumen via passive encapsulation [45]. This encapsulation effectively converts poorly soluble small molecules into uniformly dispersed nanoformulations under physiological conditions. Studies have confirmed that PDEVs outperform traditional artificial nanocarriers in certain aspects. Ginger-EVs serve as natural nanocarriers for drug encapsulation. Their natural plant-derived membrane composition improves biocompatibility, prolongs blood circulation half-life, and reduces immunogenicity relative to synthetic nanocarriers [46]. Studies have demonstrated that PDNVs can be loaded with diverse compounds (acridine orange, vinblastine BODIPY) via trains of biphasic pulses, with loading efficiency superior to commercial liposomes [47]. Combined with the aqueous solubility of PDEVs, this largely mitigates the limitations of PDNPs arising from poor water insolubility.

#### Protection from degradation and improved *in vivo* stability

Beyond solubility, free PDNPs suffer from rapid metabolic degradation and short circulation half-lives *in vivo*. Orally delivered PDNPs are easily degraded by gastric acid and digestive enzymes in the upper gastrointestinal tract. Meanwhile, intravenously injected PDNPs are rapidly cleared by the reticuloendothelial system or metabolized by hepatic enzymes, leading to low *in vivo* retention and unsatisfactory therapeutic effects [48]. The complete phospholipid bilayer membrane of PDEVs serves as a stable physical protective barrier, which can effectively shield encapsulated PDNPs from harsh physiological environments and enzymatic degradation. For oral administration, PDEVs can resist strong gastric acid and the hydrolysis of pepsin and pancreatic enzymes during gastrointestinal transit. They maintain intact structures and successfully deliver loaded PDNPs to the colonic lumen [49]. A large number of *in vitro* simulated digestion experiments have verified this protective effect. For example, ginger-derived exosome-like nanoparticles can maintain structural integrity in simulated gastric and intestinal fluids, effectively avoiding the premature release and degradation of encapsulated phytochemicals [50]. This membrane barrier function greatly prolongs the gastrointestinal residence time and blood circulation half-life of encapsulated PDNPs. It helps sustain effective therapeutic drug concentrations *in vivo* for a longer time and reduces the frequency of clinical administration, significantly improving the overall pharmacokinetic performance of plant-derived natural products. For systemic administration, ginseng-derived nanovesicles mainly accumulate in the liver and spleen following intravenous or intraperitoneal injection, and persist in the circulation for more than 48 h, indicating a relatively long systemic half-life [51]. For oral delivery, the small particle size and hydrophilic lipid surface of PDEVs enable efficient penetration through intestinal mucus layers, transport across the gastrointestinal mucosa and epithelial barriers, and resistance to gastric acid and digestive enzyme degradation [52, 53].

#### Tissue targeting and reduced off-target distribution

Nonspecific biodistribution is another key limitation of free PDNPs, leading to off-target toxicity and suboptimal drug exposure at tumor sites. The inherent tissue distribution propensity of PDEVs, combined with engineered targeting modification strategies, can significantly ameliorate the non-specific distribution of PDNPs. For systemic administration, ginseng-derived nanovesicles mainly accumulate in the liver and spleen following intravenous or intraperitoneal injection [51]. In inflamed colonic tissue or colon tumor regions, the damaged mucosal barrier exhibits increased permeability. This property enables PDEVs to preferentially accumulate at lesion sites rather than normal intestinal tissue, conferring colon-targeted delivery and prolonged retention at pathological sites [51]. Certain PDEVs possess inherent active targeting properties: mulberry-derived EVs carry MGDG, which targets ASGPR on the surface of hepatocellular carcinoma cells, enabling selective accumulation in liver tumor cells [54].

While improved delivery represents a foundational dimension of PDNPs–PDEVs synergy, the therapeutic benefits extend beyond pharmacokinetic enhancement. PDEVs also exert intrinsic antitumor activity that directly complements the pharmacological effects of PDNPs.

### Antitumor activities of PDEVs

In cancer treatment, PDEVs enhances therapeutic efficacy by inducing apoptosis, regulating the cell cycle, inhibiting tumor metastasis, and modulating the TME (Tumor Microenvironment) (Figure 2).

**FIGURE 2:**
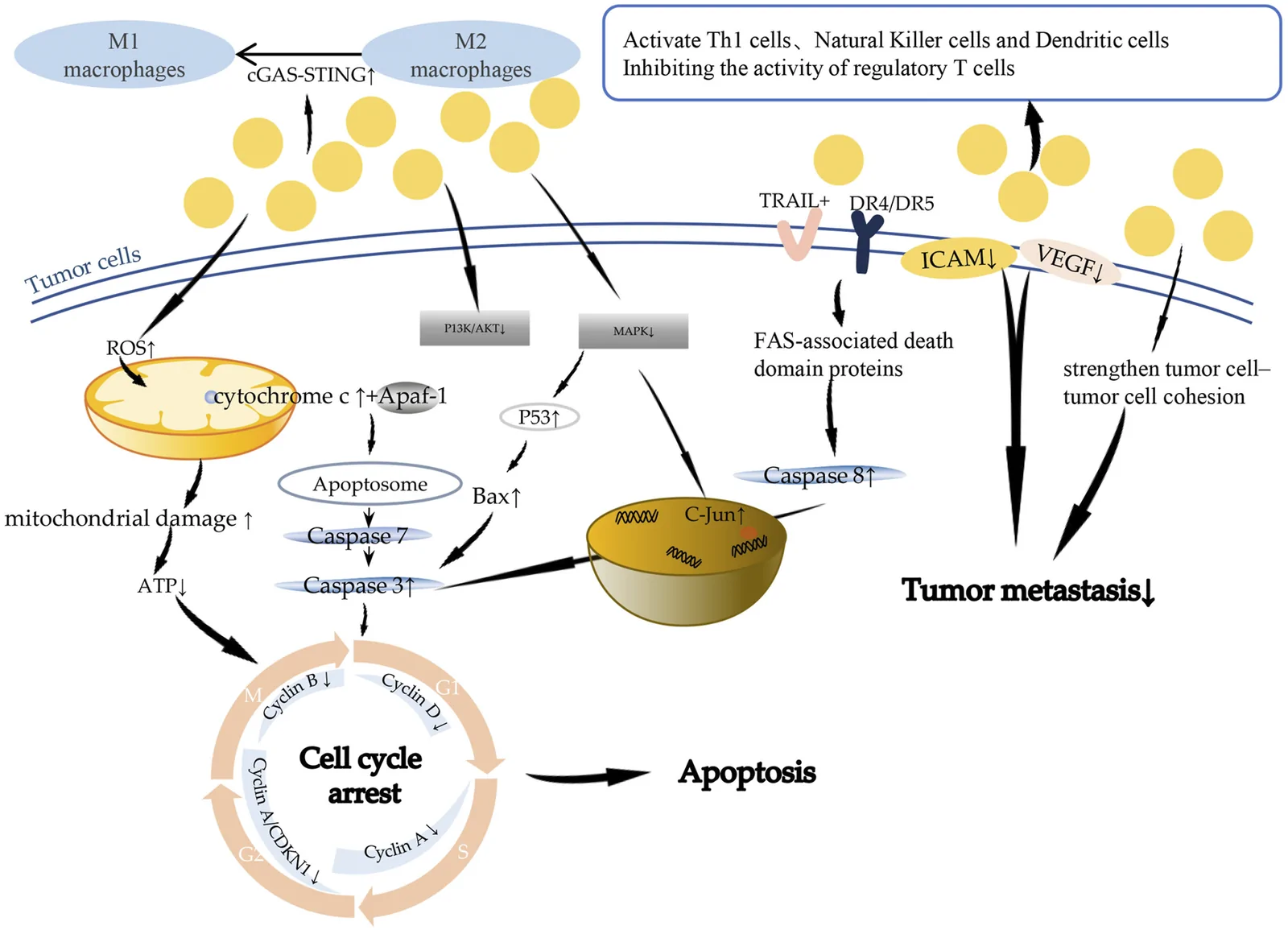
Mechanisms of PDEVs in cancer research. PDEVs induce cell cycle arrest and apoptosis. PDEVs cause mitochondrial dysfunction via reactive oxygen species (ROS) accumulation, contributing to tumor cell apoptosis. Specifically, PDEVs inhibit the PI3K/AKT and MAPK/ERK pathways, leading to upregulation of p53 expression, which subsequently blocks cell cycle progression and triggers apoptosis. PDEVs impair tumor cell migration by downregulating ICAM and VEGF expression, which reduces intercellular adhesion while strengthening tumor cell–tumor cell cohesion. PDEVs remodel the tumor immune microenvironment. They promote the polarization of tumor-associated macrophages (TAMs) from the pro-tumor M2 to the anti-tumor M1 phenotype via activation of the cGAS-STING intrinsic immune signaling pathway. Additionally, PDEVs activate Th1 cells, natural killer (NK) cells, and dendritic cells (DCs), while suppressing regulatory T cell (Treg) activity, collectively inhibiting tumor proliferation and growth.

#### PDEVs induce apoptosis in tumor cells

PDEVs induce apoptosis in cancer cells through a diverse array of interconnected signaling cascades, with the reactive oxygen species (ROS)-mitochondrial damage axis being one of the most frequently reported pathways. For instance, bitter melon-derived EVs overcame chemoresistance in oral squamous cell carcinoma by triggering ROS accumulation and mitochondrial dysfunction [55]. Similarly, *Centella asiatica*-derived EVs selectively induced ROS accumulation in HepG2 cells, leading to apoptosis without affecting normal hepatocytes [56]. In a more comprehensive *in vivo* study, *Taraxacum mongolicum*-derived EVs depolarized the mitochondrial membrane in A549 xenografts, resulting in elevated mitochondrial ROS, decreased ATP production, and tumor growth retardation, with no significant toxicity to major organs [57]. These observations collectively support a model in which mitochondrial damage facilitates cytochrome c release, apoptosome formation, and subsequent caspase cascade activation [55].

Beyond the mitochondrial pathway, MAPK and PI3K/AKT signaling are also prominently implicated. Grapefruit-derived EVs exerted anti-proliferative effects in A375 melanoma cells by co-inhibiting the PI3K/AKT and MAPK/ERK axes [11]. *Panax notoginseng*-derived nanovesicles disrupted redox homeostasis via p38-MAPK/NRF2 inhibition, triggering ferroptosis, autophagy, and PANoptosis [58] *Dipsacus-*derived EVs induced osteosarcoma cell apoptosis, potentially via p38 and JNK activation, while also demonstrating preferential tumor accumulation in optical imaging studies [59]. *Garcinia mangostana* L.-derived exosome-like nanoparticles, rich in alkaloids, flavonoids, and phenolic acids, targeted AKT1 and MAPK1 to suppress glioma cell proliferation [60]. Collectively, these findings position the MAPK/PI3K axis as a central hub for PDEV-mediated apoptosis.

Additionally, garlic-EVs significantly reduced Bcl-2 expression by approximately 60% and 65% in A498 and A549 cells, respectively. Concurrently, they upregulated pro-apoptotic genes (including caspase-3, caspase-9, and Bax) and increased p53 expression threefold [61]. Tea flowers-derived EVs exhibited a similar mechanism [62]. In addition, treatment with onion-derived nanovesicles significantly increased the number of apoptotic cells in PC-3 and HeLa cells. It also altered apoptosis-related proteins, including Bcl-2, Bax, and caspase-3, suggesting that the anticancer effects are mediated through apoptosis induction [63].

Several studies have demonstrated that PDEVs exhibit tumor-homing properties [56, 57, 59]. This may be mediated by TNF-related apoptosis-inducing ligand (TRAIL). TRAIL, as a homotrimer, binds to its homologous death receptor 4/5 on the target cell surface, leading to the recruitment of FAS-associated death domain proteins, which assemble and activate caspase-8. This activation subsequently leads to caspase-3 activation, initiating apoptosis [64]. Raimondo and colleagues validated this mechanism by using EVs derived from lemon juice-derived EVs. Their methylthiazolyldiphenyl-tetrazolium bromide assay (MTT) demonstrated that Lemon-derived EVs treatment inhibited proliferation across multiple tumor cell lines. In the CML xenograft model, the expression of TRAIL and death receptor 5 increased in tumor tissues of EVs-treated mice, while levels of pro-angiogenic factors, such as VEGF-A, IL-6, and IL-8, decreased. This finding suggests that lemon-derived EVs activate the TRAIL-mediated apoptosis pathway to effectively inhibit cancer cell proliferation [65].

In addition to these pro-apoptotic pathways, several studies have reported PDEV-mediated pro-survival or proliferative effects in non-cancerous cells, revealing a striking context-dependency. In contrast to the tumor-suppressive actions described above, citrus-derived EVs stimulated Akt phosphorylation and promoted human skin fibroblast proliferation [66]. Yam-derived exosome-like nanovesicles activated MAPK signaling and upregulated p38 to enhance MC3T3-E1 osteoblast proliferation and differentiation [67]. Moreover, citrus-EVs inhibit HT-1080 fibrosarcoma but show no effect on MCF-7 breast cancer cells, highlighting that responses are cell-type dependent [66]. These opposing outcomes highlight that the same or related signaling pathways can be differentially regulated depending on the PDEVs source, the cargo profile, and crucially, the recipient cell type. This context-dependency raises fundamental questions about the tumor selectivity of PDEV-induced apoptosis.

#### PDEVs regulate tumor cell cyclin

PDEVs can inhibit tumor cell proliferation by regulating the expression of cell cycle-related proteins, including cyclin A, B, and D. For instance, grapefruit-EVs significantly inhibit the proliferation of lung, skin, and breast cancer cells. This inhibition is closely associated with reduced expression levels of cyclin B1 and B2, along with the upregulation of cell cycle protein-dependent kinase inhibitor 1, leading to cell cycle arrest in the G2/M phase [11]. Tea-derived EVs decrease levels of cyclins A, B, and D in tumor cells, remarkably increasing the ratio of cells in the G0/G1 phase and S phase, thus inhibiting cell cycle progression [62, 68]. Mulberry-EVs benefit from MGDG, which targets the ASGPR on hepatocellular carcinoma cell surfaces. This allows mulberry-EVs to accumulate in hepatocellular carcinoma cells, blocking the cell cycle and causing arrest in the G0/G1 phase [54]. Interestingly, PDEVs not only inhibit overexpression of tumor cell cycle proteins but may also exploit this overexpression as a potential therapeutic target [62, 69].

Collectively, these studies demonstrate that PDEVs consistently downregulate cyclin expression and induce cell cycle arrest, thereby inhibiting tumor cell proliferation and migration. However, several critical observations merit discussion. First, the phase at which cell cycle arrest occurs varies across PDEVs sources—grapefruit-EVs induce G2/M arrest [11], whereas tea- and mulberry-EVs predominantly cause G0/G1 arrest [54, 62, 62]. This discrepancy may reflect differences in the specific cyclins targeted or the involvement of distinct upstream signaling pathways, but direct comparative studies are lacking. Second, the causal relationship between cell cycle arrest and apoptosis remains unclear. While both phenomena are consistently observed, most studies report them as parallel outcomes without investigating whether cell cycle dysregulation is a prerequisite for apoptosis, a consequence, or an independent event. Time-course experiments using cyclin-specific inhibitors or siRNA-mediated knockdown would be required to establish causality.

#### PDEVs inhibit tumor metastasis

In addition to its substantial role in inducing apoptosis and regulating the cell cycle in cancer cells, PDEVs have demonstrated noteworthy effects in inhibiting tumor spread. For instance, grapefruit-EVs can reduce the expression of intercellular adhesion molecule 1, which may limit the abnormal adhesion capacity of melanoma cells, thereby inhibiting their spread [11]. Garlic- EVs play a significant role in inhibiting tumor angiogenesis. Following garlic-EVs treatment, the release of VEGF from A498 and A549 cells was significantly reduced, effectively inhibiting angiogenesis and thereby limiting tumor growth and spread [61]. *Brucea javanica*-derived EVs deliver 10 functional miRNAs to 4T1 cells, inhibiting their growth and metastasis by modulating the PI3K/Akt/mTOR signaling pathway and promoting ROS/caspase-mediated apoptosis. These EVs inhibited VEGF secretion, thereby inhibiting tumor growth and demonstrating a high safety profile in a breast cancer model [12]. In addition, *Panax notoginseng*-derived nanovesicles can, on one hand, enhance the adhesion of homologous tumor cells to restrict their detachment and dissemination; on the other hand, they impair the metastatic cascade by disrupting chemokine-mediated communication and tumor-endothelial adhesion, thereby inhibiting cancer cell migration [58].

#### PDEVs modulate the tumor microenvironment

PDEVs can also enhance tumor cell killing by T cells by modulating the TME. For instance, ginseng-EVs restore antitumor T cell function by reprogramming TAMs, reducing immune checkpoint expression, and alleviating exhaustion through enhanced T cell infiltration and inhibited the activity of regulatory T cells [70, 71]. Ginseng-EVs can activate the expression of the transcription factor T-bet by regulating the arginine metabolic pathway in the TME, further suppressing immune checkpoint expression and enhancing antitumor immunity [72]. In addition, Artemisia annua-derived EVs remodel TAM into a pro-inflammatory phenotype through activation of the cGAS-STING pathway, a key innate immune pathway that triggers antitumor inflammatory responses, thereby inhibiting tumor growth [73].

Methodological limitations must be acknowledged. Most mechanistic studies rely on a narrow set of end-point assays—such as MTT, caspase activity measurements, or Bcl-2 family protein expression—without employing orthogonal confirmatory methods to rigorously confirm apoptosis. This restricts the certainty of apoptotic characterization. Moreover, functional disparities across studies may partly stem from differences in isolation protocols—ultracentrifugation may co-isolate protein aggregates that confound apoptosis readouts—yet direct comparative evidence within the same plant source is lacking. Finally, *in vivo* validation remains limited: while a few xenograft studies exist (lemon-EVs in CML; Taraxacum-EVs in A549), most mechanistic data are derived from 2D cell culture, which poorly recapitulates the tumor microenvironment. Addressing these methodological gaps will be essential to strengthen the mechanistic rigor of PDEVs apoptosis research.

## Engineering PDNPs–PDEVs delivery systems for synergistic cancer therapy

To date, more than half of clinically used nanomedicines are based on liposomes [74]. PDEVs, similar to synthetic nanoparticles, possess innate drug delivery capabilities. They possess a lipid bilayer structure similar to cell membranes, which provides high stability and biocompatibility *in vivo*. This lipid bilayer acts as a protective barrier for drugs, shielding them from degradation and enabling efficient delivery to target cells. Moreover, PDEVs have several inherent advantages over liposomes due to their natural properties: (1) PDEVs effectively avoid nanoparticle-induced clearance and adverse immune reactions due to their excellent biocompatibility [75]. Zhang and colleagues used ginger-EVs and doxorubicin for drug delivery, enhancing the EVs' functionality by functionalizing membranes with folic acid, as folic acid receptors are present in many tumor cells. They tested the cytotoxicity of ginger-EVs using assays such as MTT and electrocellular-substrate impedance sensing, observing that they were safer compared to cationic liposomes [76]. (2) Unlike synthetic nanoparticles, which serve solely as drug carriers, PDEVs contain innate bioactive components. Their payload can be used alone or in combination with active therapeutic agents. One study demonstrated that celery-derived EVs containing doxorubicin exhibited superior absorption and therapeutic activity compared to liposomes [77]. (3) PDEVs exhibit enhanced cellular uptake, with recent studies reporting over 80% internalization of PDEVs compared to 40% for liposomes [68, 78].

### Drug loading methods for PDEVs

In recent years, PDEVs have attracted extensive attention as promising candidates for nanodrug delivery systems due to their unique advantages. The efficacy of PDEVs as drug delivery systems hinges critically on the loading of therapeutic cargo. This section critically evaluates the predominant loading methodologies, weighing their respective advantages and limitations in this context (Table 4).

**TABLE 4:** Comparison of common drug loading strategies for PDEVs.

Method	Advantages	Limitations	Application
Passive co-incubation	Simple, mild to EVs, no external force, minimal membrane damage	Efficiency is significantly influenced by drug properties	• Cabbage EVs + doxorubicin, 37 °C, 4 h: no size change [144] • Lemon EVs + doxorubicin: Efficiency 18.84 ± 0.56% [80]
Electroporation	Higher loading efficiency for nucleic acids; tunable via EV/drug ratio and voltage	Potential membrane damage, compromising structural integrity, function, and *in vivo* stability/efficacy	• siRNA-loaded EVs knocked down BACE1 in mouse brain [83]
Sonication	High loading efficiency (superior to passive incubation and electroporation); can load negatively charged substances	Causes morphological changes and significant size increase, compromising membrane integrity	• Doxorubicin/paclitaxel loading achieved up to 28% (vs. 1% for passive, 5% for electroporation) [90, 91] • Macrophage EVs + paclitaxel increased cytotoxicity by > 50-fold [91]
Freeze-thawing	Mild to EVs; simple operation; higher loading efficiency than sonication	Frequent temperature changes may cause protein inactivation and size enlargement	Commonly used for animal-derived EVs (ADEVs); often combined with other methods
Extrusion	Produces EVs of uniform size (e.g., ∼200 nm) with protein composition similar to native EVs	Repeated extrusion may impair biological activity and cause loss of lipid bilayer	Often combined with freeze-thawing to prepare ∼200 nm EVs [95]
Microfluidics	Precise control	Emerging technique; requires further validation	Grapefruit EVs + siRNA: size 122 ± 32.5 nm, loading efficiency 11% [98, 98]

The most common method of loading involves passive techniques that utilize diffusive and lipophilic interactions between the therapeutic drug and the PDEVs' lipid bilayer. This process involves incubating a drug-containing solution with EVs, followed by removing the unloaded drug using ultrafiltration [79]. A separate study reported that a loading efficiency of 18.84 ± 0.56% was achieved during co-incubation of doxorubicin with lemon-derived EVs [80]. Nemidkanam et al. co-incubated clarithromycin with PDEVs derived from Malpighia emarginata at the ratio of 1:1. The system achieved a high encapsulation efficiency (EE) of 92.10%, while exhibiting a low loading capacity (LC) of only 1.60% [81]. Co-incubation is a passive process lacking additional forces to promote diffusion, typically resulting in relatively low loading efficiency. Optimization of incubation parameters, including incubation duration, temperature, pH, and the ratio of drug to EVs, is critical for elevating loading efficiency. Furthermore, mild stirring or sonication applied during co-incubation can further boost the drug loading performance of EVs [82]. Drug physicochemical properties also greatly affect loading efficiency. This factor severely restricts the loading of hydrophilic macromolecular drugs. PDEVs have a strongly negative surface charge, with a zeta potential of −10 to −25 mV. They can adsorb positively charged drugs directionally through electrostatic interaction. This mechanism effectively improves the loading efficiency of cationic drugs [76]. Drugs with different properties require tailored loading strategies: hydrophobic small-molecule drugs can directly penetrate the EVs membrane and achieve successful loading through simple co-incubation; in contrast, hydrophilic compounds cannot spontaneously cross the lipid vesicle membrane, thus requiring active interventions to facilitate their entry through pores on EVs. Techniques including electroporation, sonication, extrusion, and freeze-thaw function by generating pores of sufficient size on the EV surface to allow the internalization of drug molecules.

Electroporation is commonly used to load small molecules such as siRNA, antisense oligonucleotides. siRNA was successfully loaded into EVs by electroporation and achieved specific knockdown of the BACE1 gene in the mouse brain, which can be used to improve the loading efficiency by adjusting the EVs: drug ratio and the supplied voltage [83]. In ADEVs, electroporation is one of the most commonly used methods for loading miRNA [45]. Multiple studies was calculated as 17%–25% of the initial nucleic acid used for loading [7], indicating that electroporation was relatively efficient. The underlying principle of this technique can be transferred to PDEVs; however, direct empirical data specific to PDEVs remain limited. Rabienezhad et al. first employed electroporation for siRNA loading into PDEVs, achieving a transfection efficiency of 13%—comparable to that reported for ADEVs—after co-incubation and sonication proved ineffective [84]. This demonstrates that electroporation, although originally optimized for mammalian EVs, is applicable to PDEVs for nucleic acid delivery. Nevertheless, reported loading efficiencies vary considerably depending on the PDEVs source and cargo type: dsRNA loading in citrus-derived PDEVs reached only 6.0% [85], whereas CX5461 encapsulation in *Sophora flavescens*-derived PDEVs achieved 23% [86]. Other studies have demonstrated that by optimizing electroporation parameters—such as a nanovesicle-to-drug concentration ratio of 1:2 or 1:4—the drug loading efficiency of PDEVs can be enhanced to 70%–80% [87]. However, electroporation may disrupt the integrity of the EVs' membrane, leading to impaired structure and function of the EVs [88]. This may affect the stability and biocompatibility of EVs, which in turn may affect their delivery efficiency and therapeutic efficacy *in vivo*.

Sonication is a way to load drugs into the PDEVs using ultrasound energy to disrupt the EVs' lipid bilayer with higher efficiency than electroporation. Compared with co-incubation, sonication achieves better drug loading efficiency. Huang et al. loaded curcumin into ginger-derived PDEVs by 3 min of sonication. The feeding mass ratio of curcumin to PDEVs was 1:1. This protocol reached an LC of 94% and an EE of 89% [89]. The internalization of doxorubicin and paclitaxel using multiple sonication cycles interspersed with cooling periods, followed by incubation at 37 °C, achieved an efficiency of up to 28%, compared to 1% for passive incubation and 5% for electroporation [90, 91]. Kim et al. used an ultrasonic bath to load paclitaxel into macrophage-released EVs, which resulted in an increase in cellular cytotoxicity by more than 50-fold and had potent anticancer effects in this mouse model [91]. These methods show higher encapsulation efficiency than co-incubation. Moreover, sonication can be used for the loading of negatively charged substances. However, many studies prove sonication damages the pharmacological activity of PDEVs. Cao et al. found that sonication weakened the ability of ginseng-derived EVs to induce macrophage polarization [72]. The Atomic Force Microscope (AFM) images of sonicated EVs demonstrated non-spherical aggregates with a variety of shapes, which led to a significant increase in size (100.5 ± 13.5 nm to 179.0 ± 10.6) [90]. Li et al. treated PDEVs from *Curcumae Longae* Rhizoma, *Polygonati* Rhizoma and *Gastrodiae* Rhizoma with probe sonication. They excluded chemical damage caused by ultrasound. The treated PDEVs displayed reduced antioxidant activity and cellular uptake ability to different extents [92]. All these results suggest that intact PDEVs structure is vital to their biological functions.

Freeze-thawing is commonly used for ADEVs loading, where a drug solution is mixed with the EVs, rapidly frozen at −80 °C, and then thawed in a water bath at 30 °C, and the drug can be loaded into the EVs as the number of cycles increases. For example, tomato-derived EVs achieved a calcitriol encapsulation efficiency of 34.8% after three freeze-thaw cycles alternating between −80 °C and 4 °C [93]. The freeze-thaw method adopts relatively mild processing conditions. It avoids chemical reagents and intense physical treatment, which effectively preserves drug bioactivity. Thus, this strategy is particularly suitable for thermally unstable drugs. Additionally, freeze-thaw cycling provides a higher loading rate than sonication, but frequent temperature changes may lead to protein inactivation and an increase in EVs size [94].

To obtain EVs of uniform size, extrusion, and microfluidics are optional loading methods. For example, Fuhrmann used a combination of freeze-thawing and extrusion to prepare EVs with a size of about 200 nm, which have similarity to natural EVs in terms of size and protein content [95]. Chen combined sonication and extrusion strategies to load doxorubicin (DOX) into PDEVs. This approach increased the encapsulation efficiency nearly 10-fold compared with other conventional methods [96]. However, the repetitive coextrusion process may impair the biological activity of PDEVs [97]. Loss of the lipid bilayer on the membrane is also an important issue that cannot be ignored. A new method of EVs drug loading-microfluidics-has been reported recently. Loading siRNA into grapefruit-EVs yielded nanoparticles with a size of 122 ± 32.5 nm, which achieved a loading efficiency of 11% and accomplished intracellular delivery of siRNA and gene inhibition in HaCaT cells [98].

Overall, PDEVs outperform artificial liposomes in multiple aspects, including higher drug loading efficiency, favorable biocompatibility, low immunogenicity, cost-effectiveness and inherent targeting capacity [52]. Nevertheless, most active loading strategies such as sonication and electroporation can improve encapsulation efficiency yet impair the intrinsic biological activity of vesicles to varying degrees. PDEVs exhibit inherent variations in physicochemical properties and compositions due to factors like harvest season and plant health status, which poses great challenges to the standardization of their particle size and internal contents. Accordingly, establishing standardized protocols for extraction, characterization and storage is critical to generate consistent results applicable to clinical research. Apart from that, selecting suitable loading procedures to construct reproducible delivery systems and precisely regulate pharmacological effects remains an urgent research concern. Although some studies have proposed to utilize indocyanine green to adsorb salt ions in PBS and induce hypotonic stress to cause Aloe vera cells to secrete Aloe vera-derived EVs enriched with long carbon chain steroid esters, PC, phosphatidylinositol, and ceramides, which enhances the rigidity and methodical stability of EVs derived from Aloe, such studies are mostly in the exploratory stage, and their safety and efficacy have yet to be verified [99].

### Engineering modification strategies for enhanced targeting

Although previous studies have demonstrated some specificity of PDEVs for tumor cells, the mechanisms driving their cellular internalization and cargo delivery to the recipient cells remain poorly understood. It remains unclear whether PDEVs internalization occurs via a nonspecific process, such as macropinocytosis, or through a specific receptor-dependent pathway. Despite PDEVs sharing a common surface proteome, it is also uncertain whether these surface proteins are responsible for the targeting effect. It has been suggested that their induction of apoptosis in tumor cells occurs mainly due to PDEVs’ natural biodistribution [100, 101].

To overcome the limited challenge of their native targeting mechanisms, engineering the surface of PDEVs has emerged as a key strategy to achieve precision therapy. This approach aims to enhance targeting efficacy, improve drug delivery, and reduce off-target effects. Folic acid is a commonly used targeting ligand, as the folic acid receptor is highly expressed in many cancer cells but has limited distribution in normal organs [102]. Utilizing RNA nanotechnology, researchers designed Arrow-tail RNA nanoparticles and modified them with cholesterol to enable binding to ginger-EVs membranes. This modification displays folic acid ligands on the EVs' surface, enhancing ginger-EVs binding and uptake into KB cells, which facilitates more efficient siRNA delivery to tumor tissues and specific gene silencing [103].

Another common strategy is to immobilize molecules such as cyclic arginine-glycine-aspartic acid (cRGD) on the EVs' surface. cRGD recognizes and competitively binds to αvβ3 integrins, highly expressed on the surface of many solid tumors [104], which can also block tumor cell adhesion to the extracellular matrix or directly induce apoptosis. Exploiting these features, Chen et al. modified lemon-derived EVs with cRGD using an EDC/NHS chemical coupling method, thereby increasing EVs' accumulation at the tumor site [105]. Niu et al. first linked pH-sensitive adipic dihydrazide to doxorubicin to promote its release in an acidic TME. They then coupled adipic dihydrazide-doxorubicin and cRGD to the carboxyl group of heparin, which was subsequently linked to the active amino group on the grapefruit-EVs membrane to create a new delivery platform that targets gliomas and controls drug release [106].

Click chemistry is a prominent technique for the biofixation of targeted ligands to the surface of EVs through covalent bonding. This technology has been demonstrated in ADEVs. To modify EVs, molecules containing azide or alkyne groups are added to the parental cell culture medium, introducing corresponding reaction groups on biomolecules like glycans or proteins on the EVs membrane surface. Various targeting molecules can then be conjugated to these reaction groups using click chemistry, enabling precise modification of EVs [107]. For instance, azide-labeled αvβ3 integrin-specific peptide RGDyK [108] and glioma-targeted peptide RGE [109] have been successfully demonstrated on EVs surfaces. However, a key limitation of alkyne modifications is the lack of site-specific control. Consequently, click chemistry may disrupt the structure and function of EVs’ proteins. This trade-off between labeling efficiency and functional integrity remains a central challenge for covalent modification strategies.

### Applications of PDEVs as a delivery system for PDNPs in cancer therapy

The preceding section has outlined how PDEVs improve the bioavailability, stability, and tumor targeting of PDNPs. This section focuses on the therapeutic evidence, examining how PDEVs-mediated delivery translates into improved anticancer efficacy for representative PDNPs (Table 5).

**TABLE 5:** Summarized list of PDNPs used in PDEVs-mediated delivery.

Source	Target	PDNPs	Other drugs	References
Ginger	Cancer	Folic acid	Doxorubicin	[76]
	Colon cancer	Gingerol, shogaol	miRNA	[69]
	Ulcerative colitis	6-Shogaol	N/A	[121]
Grapefruit	Colon cancer, breast cancer	Curcumin	Doxorubicin	[145]
	Glioma	Paclitaxel, folic acid	JSI-124,siRNA	[106]
	Brain tumor, colon cancer	Paclitaxel, folic acid	JSI-124	[78]
Grape	N/A	Curcumin	N/A	[146]
	Molt-4 cells	Fisetin	N/A	[147]
Broccoli	Colon cancer	Sulforaphane	N/A	[120]
Sesame leaf	Gastrointestinal tract	Luteolin	N/A	[122]

Curcumin, a natural polyphenolic compound, is the principal bioactive constituent of turmeric (*Curcuma longa*). Preclinical studies conducted over the past three decades have demonstrated curcumin’s extensive therapeutic benefits. Curcumin exhibits the following pharmacological activities: antitumor, antifibrotic, antioxidant, and antimicrobial effects. It may mitigate the progression and metastasis of leukemia, prostate, colon, breast, and several other cancers [110, 111]. However, curcumin exhibits poor transmembrane permeability and low bioavailability. Clinical trials have shown that even at very high doses of 8–12 g per day, plasma concentrations of curcumin remain low [112].PDEV encapsulation substantially addresses this limitation. In preclinical mouse models of colon and breast cancer, PDEVs-encapsulated curcumin exhibited markedly improved bioavailability, resulting in enhanced tumor growth inhibition and prolonged survival compared with free curcumin [113]. Early clinical evidence supports this translational potential: in the phase I/II trial NCT01294072, plant-vesicle-mediated delivery increased curcumin accumulation in colon tumor tissue relative to normal colonic mucosa, with an acceptable safety profile. Notably, the interaction between orange-derived EVs and curcumin extends beyond simple pharmacokinetic rescue. The two components form a bidirectional antioxidant network: encapsulation increases curcumin bioavailability by 5–6-fold, while curcumin dose-dependently enhances the antioxidant capacity of the EVs themselves [114].

Moreover, grapefruit-EVs have been employed for the oral delivery of curcumin, effectively overcoming its poor oral bioavailability [115]. Additionally, researchers have loaded curcumin into mouse macrophages derived EVs and coupled EVs membranes with neuropilin-1 targeting peptides, enabling EVs to target gliomas. These EVs crossed the blood-brain barrier efficiently, providing promising results for targeted imaging and treatment of gliomas [109]. Paclitaxel is a natural secondary metabolite isolated from the bark of the Pacific yew. It represents a cornerstone in the treatment of several prevalent malignancies, including breast, ovarian, and non-small cell lung cancers. To enable intravenous administration, paclitaxel must be solubilized using a mixture of Cremophor EL, ethanol as solvents. Unfortunately, these excipients often induce severe allergic reactions and peripheral neuropathy, which frequently limit both the duration of treatment and therapeutic efficacy [116]. While Albumin paclitaxel (Nab-paclitaxel) improves solubility and safety, it still has a 34% rate of adverse reactions [116–118]. This high drug-loading capacity, combined with the innate biocompatibility and targeting potential of PDEVs, positions them as a promising alternative to synthetic nanoparticle platforms. They hold the potential to enable oral administration[119], thereby improving patient compliance and quality of life—a significant advantage over current intravenous formulations.

Beyond these well-characterized compounds, PDEVs serve as a versatile platform for a broader range of bioactive PDNPs with inherent bioavailability challenges. For example, gingerols from ginger, sulforaphane from broccoli, and luteolin from perilla leaves have all been successfully loaded into PDEVs, with preliminary studies demonstrating enhanced anticancer efficacy in tumor models [120–122]. These examples underscore the generality of the PDEV platform in overcoming key pharmaceutical limitations that have long hampered the clinical translation of plant-derived natural products.

Currently, research on PDEVs is predominantly preclinical, with only 5 registered clinical trials related to PDEVs (Table 6). Nevertheless, accumulating preclinical evidence underscores the considerable potential of PDEVs as multifaceted anticancer agents. The key challenges that remain—including standardization of isolation and characterization, understanding of *in vivo* pharmacokinetics and biodistribution, and comprehensive safety assessment.

**TABLE 6:** Clinical trials related to PDEVs.

Source	NCTID	Trial description	Year	Adaptation disease	Outcome
Grape	NCT01668849	Preliminary clinical trial investigating the ability of plant exosomes to abrogate oral mucositis induced by combined chemotherapy and radiation in head and neck cancer patients	2012	Oral mucositis related to tumor treatment	N/A
N/A	NCT01294072	Phase I clinical trial investigating the ability of plant exosomes to deliver curcumin to normal and malignant colon tissue	2011	Colon cance	N/A
Aloe, ginger	NCT03493984	A preliminary clinical trial investigating the ability of plant exosomes to mitigate insulin resistance and chronic inflammation in patients diagnosed with polycystic ovary syndrome	2018	Polycystic ovary syndrome	N/A
Ginger	NCT04879810	Pilot clinical trial investigating the ability of plant exosomes +/- curcumin to abrogate symptoms of inflammatory bowel disease	2021	Irritable bowel disease	Change in inflammation on colonoscopy (decrease in inflammatory cells in the biopsy after treatment versus before treatment)
Lemon	NCT04698447	Effects of the natural supplement CitraVes containing nanovesicles delivered from citrus limon (L.) juice on cardio-metabolic risk factors in subjects with metabolic syndrome	2021	Metabolic syndrome	N/A

## Discussion

### Advantages of PDNPs–PDEVs nanoplatforms

The examples presented above collectively demonstrate the broad generality of PDEV-based platforms in overcoming key pharmaceutical limitations that have long hampered the clinical translation of PDNPs. This broad applicability rests on several distinct advantages relative to alternative delivery systems. First, PDEVs offer a superior safety profile compared with ADEVs. ADEVs carry inherent risks of zoonotic pathogen transmission and unwanted gene or protein transfer, concerns that are largely absent with plant-derived vesicles. Second, and more fundamentally, PDEVs differ from ADEVs in their therapeutic role. ADEVs typically function as inert signaling carriers or passive delivery platforms, whereas PDEVs are intrinsically bioactive. This means PDNP–PDEV nanoplatforms derive therapeutic value from both the delivery function and the inherent bioactivity of the vesicle itself, creating a dual-action system that cannot be replicated by carrier-only platforms.

These advantages notwithstanding, the intrinsic bioactivity of PDEVs also introduces unique challenges. The complex and variable composition of PDEVs across plant sources complicates precise mechanistic attribution and batch-to-batch reproducibility. Furthermore, the lack of plant-specific EV markers and standardized characterization guidelines poses a significant barrier to quality control and regulatory acceptance. Collectively, PDNP–PDEV nanoplatforms represent a highly promising but still maturing therapeutic strategy.

### Mechanisms of PDNPs–PDEVs synergy

A foundational challenge in PDNPs–PDEVs combination therapy research lies in moving beyond phenomenological synergy outcomes to defining the underlying molecular mechanisms. PDEVs are compositionally complex, heterogeneous entities, carrying diverse bioactive molecules such as specific miRNAs, lipids, proteins, and unique plant secondary metabolites. Consequently, it remains unresolved whether their synergistic effects with PDNPs stem from PDEVs' single dominant bioactive component or from sophisticated cooperative interactions between PDNP cargo and PDEV-derived bioactive molecules.

Future mechanistic research should be structured along two axes: direct cytotoxic synergy and combined immunomodulation. Regarding direct effects, initial studies have begun to attribute PDEVs’ bioactivity to specific cargo. For instance, ginger-EVs carry compounds like 6-gingerol and 6-shogaol, which possess known anti-inflammatory and pro-apoptotic properties [123]. More directly, miRNA profiling has identified functional molecules such as ginseng-derived miR-396f, which suppresses glioma by targeting the oncogene c-MYC [123]. A key but largely unexplored direction is to elucidate how PDEVs-delivered PDNPs and intrinsic PDEVs payloads jointly enter cancer cells—whether via membrane fusion, endocytosis, or receptor-mediated uptake—and how they synergistically modulate intracellular pathways.

Beyond directly killing tumor cells, PDEVs may exert significant antitumor effects by reshaping the TME. This immunomodulatory potential involves key immune cell populations. For example, preliminary studies have demonstrated that ginseng-derived EVs can reprogram pro-tumor M2-type TAM into anti-tumor M1 phenotypes [70]. Concurrently, artemisia-derived EVs have been reported to promote the transition of macrophages toward a pro-inflammatory state [73]. Regarding adaptive immunity, ginseng-EVs demonstrate the ability to promote T cell differentiation toward CD8^+^ T cells while suppressing the abundance of regulatory T cells [70]. When combined with immunomodulatory PDNPs, these PDEV-driven effects may be further potentiated, strongly supporting the potential application of PDNPs–PDEVs combinations in tumor immunotherapy.

However, the systemic regulatory mechanisms of PDEVs-PDNPs in the TME remain far from fully understood. Key priorities include: (1) elucidating how the combination system targets and modulates other core immune cells. Important open questions include whether PDNPs–PDEVs combinations can alleviate T cell exhaustion or act as natural adjuvants to promote dendritic cell maturation; (2) identifying which specific components (e.g., particular miRNAs or lipids); drive these immunoregulatory functions; and (3) integrating multi-omics data with AI-based network modeling to predict synergistic *in vivo* efficacy, laying the foundation for precision PDNPs–PDEVs-based combination immunotherapies.

### Standardization of preparation and quality control for combination systems

PDNPs are typically well-characterized small-molecule drugs with established quality control standards. The primary analytical challenges for PDNPs–PDEVs combination systems therefore lie on the PDEV side and on the consistency of the drug-loading process.

A foundational issue confounding the field is terminological imprecision. The term “exosome” has been widely used interchangeably with EVs in both animal and plant research [124, 125]. MISEV2023 has clearly recommended using “Extracellular Vesicles” as the unified term to enhance precision and consistency across studies. The guidelines emphasize that subtype-specific terms such as “exosomes” should only be used when there is rigorous and sufficient evidence to confirm the subcellular origin and biogenesis mechanism of the vesicles. This distinction has direct implications for characterization standardization and quality control of PDNPs–PDEVs formulations.

Beyond nomenclature, the characterization of PDEVs faces substantial methodological challenges. Current practice—combining size distribution, zeta potential, TEM, NTA, and Western blot marker validation—remains largely qualitative and lacks sufficient accuracy for purity assessment, with subcellular origin remaining largely undetermined. To date, only a few candidate markers (such as PEN1 and TET8 in Arabidopsis) have been reported [126]. Nevertheless, their evolutionary conservation in other plant species has not been extensively validated. Emerging technologies like high-resolution mass spectrometry and single-vesicle proteomics enable systematic screening of PDEVs-enriched proteins. Establishing a standardized quality control system based on specific markers is essential for batch consistency and clinical translation. We strongly recommend using “EVs” or “nanoparticles” for particles whose subcellular origin has not been defined. If the use of unconfirmed terms is deemed unavoidable, they should be clearly and prominently defined at the beginning of each publication. Moreover, PDEVs heterogeneity can cause significant batch-to-batch variations in bioactivity, which in turn leads to variable synergistic outcomes when combined with PDNPs. Given the immunomodulatory potential of PDNPs–PDEVs combinations in cancer therapy, standardized *in vitro* immunological bioassays—such as quantifying macrophage polarization, dendritic cell maturation, or T-cell proliferation—should be established as core quality metrics [127].

PDEVs have a clear advantage over mammalian EVs for combination therapy development. They are derived from plants. This allows large-scale production through agriculture. For PDNPs, large-scale synthesis or extraction processes are already well established at the industrial level, meaning the main scalability bottleneck for combination formulations is the production and standardization of PDEVs, not the drug component. However, raw materials pose contamination risks (e.g., pathogens, pesticides) and seasonal variability, which may compromise PDEVs purity and function and ultimately affect the consistency of PDNPs-loaded formulations.

### Human - relevant safety

Multiple studies have reported favorable biocompatibility of PDEVs. However, comprehensive preclinical safety data for PDNPs–PDEVs combination formulations are still scarce. While the edible origin of PDEVs suggests inherent biocompatibility, this must not be equated with safety at therapeutic doses, especially via non-oral routes [128]. For instance, mulberry-derived EVs induced significant AST elevation upon intravenous administration, whereas oral delivery did not [54], underscoring that route-specific toxicology is paramount for both free PDEVs and PDNPs-loaded formulations.

A key unresolved safety concern lies in the uncharacterized immunogenicity profile of PDNPs–PDEVs combinations. Plant-specific proteins carried by these nanovesicles may introduce novel antigenic epitopes that are not encountered in mammalian extracellular vesicles, and the potential for hypersensitivity reactions upon repeated dosing has not been systematically evaluated. Furthermore, encapsulation of PDNPs may alter the physicochemical surface properties of PDEVs and modify their intrinsic immunogenicity profile. High-throughput immunological assessment platforms, including human peripheral blood mononuclear cell (PBMC)-based immunoassays and microphysiological organ-on-a-chip models, are urgently required to rigorously quantify and de-risk hidden immunogenicity hazards of the combination system.

Metabolic kinetics and long-term systemic safety represent additional critical unaddressed research gaps for PDNPs–PDEVs formulations. Following systemic administration, over 80% of plant nanovesicles accumulate in the liver and spleen, with less than 5% reaching intended target tissues [129]. This uneven biodistribution raises legitimate concerns about off-target hepatotoxicity and unintended, chronic immunostimulation in the reticuloendothelial system—concerns that are further compounded when the vesicles carry pharmacologically active PDNPs. While prior work has reported no significant AST elevation after oral PDEVs delivery [54], relying solely on a single enzymatic biomarker carries inherent analytical limitations and cannot fully exclude subtle, subclinical liver injury. Furthermore, the long-term biodistribution patterns and chronic toxicity profiles of PDNPs-loaded PDEVs remain entirely uncharacterized, representing a major barrier to clinical translation.

Batch-to-batch variability represents a critical but often overlooked issue for combination product development. Few systematic studies have characterized PDEVs harvested from separate batches of plant feedstocks, constituting a prominent barrier to industrial scale-up. Differences in plant source, growth conditions, harvest timing, and isolation protocols lead to substantial heterogeneity in PDEVs composition and bioactivity, yet the impact of such variability on both the delivery efficiency and safety profile of PDNPs–PDEVs formulations has not been systematically evaluated. Collectively, these gaps underscore that current *in vivo* evidence, while encouraging, is insufficient to support clinical translation of PDNPs–PDEVs combination therapies.

Given these uncertainties, we advocate for a toxicology-first research framework, analogous to the standardized evaluation strategies established for synthetic nanomedicines, to systematically define the full risk profile of PDNPs–PDEVs therapeutic combinations [128].

### A translational roadmap for PDNPs–PDEVs-based therapeutics

To bridge the gap between preclinical promise and clinical application, the translational roadmap for PDNPs–PDEVs combination therapeutics (Figure 3) must prioritize a “Quality by Design” paradigm, addressing three sequential and interconnected levels. (1) Raw Material Standardization (GACP). Agricultural sources are inherently variable—differing in cultivar, growth conditions, and harvest timing—and carry contamination risks (pesticides, pathogens). These variables directly affect PDEVs yield, cargo and loading efficiency for PDNPs, yet most studies provide minimal source metadata. Implementing GACP is therefore the non-negotiable foundation for batch-to-batch consistency of the final combination product. This includes standardized cultivation protocols, documented harvest windows, and validated storage conditions—prerequisites that must be established before any downstream manufacturing can be meaningfully standardized. (2) Manufacturing Scalability. Current ultracentrifugation methods are not GMP-compatible for large-scale production due to low throughput and high variability. The future lies in scalable technologies such as tangential flow filtration (TFF) coupled with size-exclusion chromatography (SEC) to achieve high yield and purity [130]. However, plant-specific challenges—membrane fouling by polysaccharides and shear sensitivity—require systematic optimization across species, and drug loading processes must be integrated into scalable manufacturing workflows to ensure consistent PDNPs encapsulation. (3) Regulatory Compliance (CMC). PDNPs–PDEVs combinations occupy a regulatory grey zone: they are neither conventional small-molecule drugs nor typical biosimilars. A robust Chemistry, Manufacturing, and Controls (CMC) package must include: identity (lipidomic/proteomic fingerprints), purity (removal of non-vesicular contaminants), potency (mechanism-linked bioassays), and stability profiles [128]. These parameters should be established early to guide process development and de-risk translation.

**FIGURE 3:**
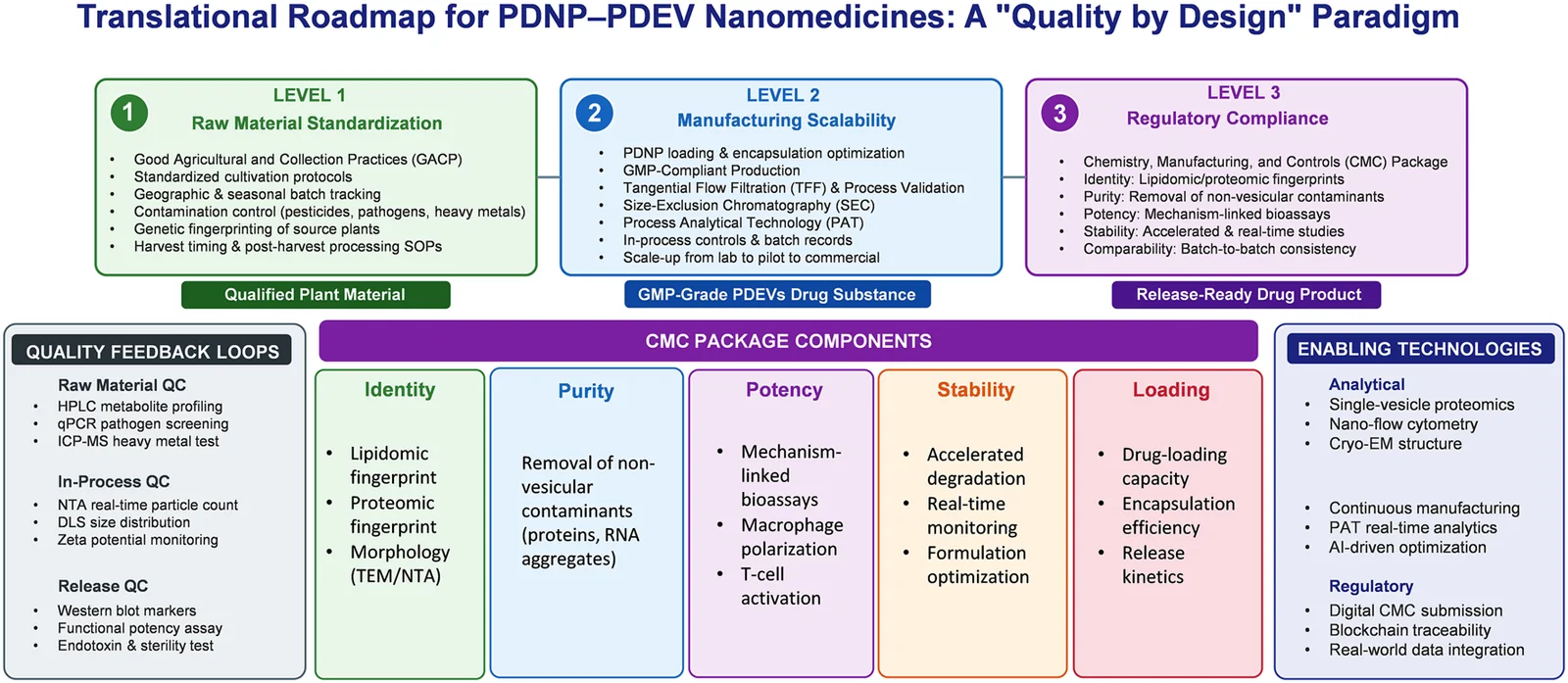
Translational Roadmap for PDNP–PDEV Nanomedicines: A “Quality by Design” Paradigm. Three sequential levels—raw material standardization, manufacturing scalability, and regulatory compliance—translate the synergistic PDNP–PDEV axis into a clinical product, with PDNP loading optimized during manufacturing. The central CMC framework comprises five dimensions: identity, purity, potency, stability, and loading capacity. Quality feedback loops and enabling technologies ensure consistent PDEV quality throughout the pipeline. SOPs, Standard Operating Procedures; GMP, Good Manufacturing Practice; QC, Quality Control; HPLC, high-performance liquid chromatography; qPCR, quantitative polymerase chain reaction; ICP-MS, inductively coupled plasma mass spectrometry; NTA, nanoparticle tracking analysis; DLS, dynamic light scattering; Cryo-EM, Cryogenic Electron Microscopy; AI: Artificial Intelligence.

Collectively, these three pillars—source control, scalable manufacturing, and regulatory-ready characterization—provide a coherent, phased framework for translating PDNPs–PDEVs-based combination therapeutics from bench to bedside. Their implementation will require coordinated efforts across academic, industrial, and regulatory stakeholders, and should be pursued in parallel rather than sequentially, as each pillar informs and constrains the others.

## Conclusion

In summary, this review has systematically examined the synergistic application of PDNPs and PDEVs in cancer therapy. PDEVs offer distinct advantages over synthetic nanoparticles and ADEVs in scalability, biocompatibility, and safety, making them particularly well-suited as both delivery vehicles and complementary bioactive agents for PDNPs-based cancer treatment. Specifically, PDEVs not only improve the solubility, stability, bioavailability, and targeted delivery of PDNPs, but also exert complementary antitumor effects through their intrinsic molecular activities, collectively leading to enhanced therapeutic efficacy and reduced systemic toxicity.

Despite these advantages, clinical translation of PDNPs–PDEVs combination strategies faces three core challenges: lack of standardized preparation/characterization protocols, incomplete understanding of synergistic mechanisms, and insufficient long-term safety data. Uncovering the molecular basis of their synergistic actions and resolving these standardization and safety gaps will be the defining steps to advance this combination therapy from bench to bedside.

## Data Availability

The original contributions presented in the study are included in the article/supplementary material, further inquiries can be directed to the corresponding authors.
